# Comparative genome-wide characterization leading to simple sequence repeat marker development for *Nicotiana*

**DOI:** 10.1186/s12864-018-4878-4

**Published:** 2018-06-27

**Authors:** Xuewen Wang, Shuai Yang, Yongdui Chen, Shumeng Zhang, Qingshi Zhao, Meng Li, Yulong Gao, Long Yang, Jeffrey L. Bennetzen

**Affiliations:** 1 Germplasm Bank of Wild Species, Kunming Institute of Botany, Chinese Academy of Sciences, 132 Lanhei Road, Kunming, 650201 People’s Republic of China; 2 Department of Genetics, University of Georgia, Athens, GA 30602 USA; 3 Agricultural Big-Data Research Center, College of Plant Protection, Shandong Agricultural University, Tai’an, 271018 China; 4 Biotechnology and Germplasm Resources Institute, Yunnan Academy of Agricultural Sciences, Kunming, 650223 People’s Republic of China; 5 Tobacco Breeding Center, Yunnan Academy of Tobacco Agricultural Sciences, Kunming, 650021 Yunnan China

**Keywords:** Genotyping technology, Marker database, Marker polymorphism, SSR, Tobacco

## Abstract

**Background:**

Simple sequence repeats (SSRs) are tandem repeats of DNA that have been used to develop robust genetic markers. These molecular markers are powerful tools for basic and applied studies such as molecular breeding. In the model plants in *Nicotiana* genus e.g. *N. benthamiana,* a comprehensive assessment of SSR content has become possible now because several *Nicotiana* genomes have been sequenced. We conducted a genome-wide SSR characterization and marker development across seven *Nicotiana* genomes.

**Results:**

Here, we initially characterized 2,483,032 SSRs (repeat units of 1–10 bp) from seven genomic sequences of *Nicotiana* and developed SSR markers using the GMATA® software package. Of investigated repeat units, mono-, di- and tri-nucleotide SSRs account for 98% of all SSRs in *Nicotiana*. More complex SSR motifs, although rare, are highly variable between *Nicotiana* genomes. A total of 1,224,048 non-redundant *Nicotiana* (NIX) markers were developed, of which 99.98% are novel. An efficient and uniform genotyping protocol for NIX markers was developed and validated. We created a web-based database of NIX marker information including amplicon sizes of alleles in each genome for downloading and online analysis.

**Conclusions:**

The present work constitutes the first deep characterization of SSRs in seven genomes of *Nicotiana,* and the development of NIX markers for these SSRs. Our online marker database and an efficient genotyping protocol facilitate the application of these markers. The NIX markers greatly expand *Nicotiana* marker resources, thus providing a useful tool for future research and breeding. We demonstrate a novel protocol for SSR marker development and utilization at the whole genome scale that can be applied to any lineage of organisms.

The Tobacco Markers & Primers Database (TMPD) is available at http://biodb.sdau.edu.cn/tmpd/index.html

**Electronic supplementary material:**

The online version of this article (10.1186/s12864-018-4878-4) contains supplementary material, which is available to authorized users.

## Background

Simple sequence repeats (SSRs), otherwise known as short tandem repeats (STRs) or microsatellites, are abundant and broadly distributed in eukaryotic and prokaryotic genomes. The length of an SSR shows extensive intra- and interspecific variation, primarily because of high rates of DNA replication error within SSRs [1–3]. Hence, SSRs are widely used for designing PCR-based markers that can be very useful for population genetic characterizations, genome mapping, and such applications as tagging trait-associated genes during marker-assisted selection [4]. The genomic quantity and distribution of SSRs differs between dicot and monocot plants [5]. Analyses of SSR distribution revealed that di-nucleotide repeats are more common than tri-nucleotide repeats in dicots, and the most frequent motifs are AT and ATT/AAT [6]. In monocot grass genomes, the most abundant motif is GA/TC, the A/T monomer and then the GCG/CGC trimer [7]. Recent analyses found that the SSR abundance increases linearly with genome size in fully assembled grass genomes [7]. Most SSRs are in intergenic regions and in 5’-UTRs [5, 6]. Characterization of the SSRs across an entire genome provides the foundation for comprehensive SSR marker development. In fact, genome-wide SSR markers have been designed for several plant species after their genome sequences were released, including in rice [8], soybean [9], *Brachypodium* [10], maize [11], foxtail millet [12], *Brassica* [13] and cotton [14].

*Nicotiana*, a member of the *Solanaceae* family, is one of the most important research model plants and is of high agricultural value worldwide [15, 16]. Species in the genus *Nicotiana* have large genome sizes, averaging ~ 2.5 Gb for diploids and ~ 4.5 Gb for tetraploids, which made it challenging to sequence whole genomes [17]. With recent advances in next generation sequencing (NGS) technologies, extensive genome sequence assemblies were generated for seven *Nicotiana* species or varieties and made available to the public [17]. These sequences are derived from the diploids *N. otophora* (*N. oto*), *N. sylvestris* (*N. syl*) and *N. tomentosiformis* (*N. tom*) and from four allotetraploids, namely *N. benthamiana* (*N. ben*) and three major commercial varieties of *N. tabacum* (*N. tab*): TN90, K326, and BX [18–21]. The *N. syl* and *N. tom* lineages diverged about 15 million years ago [22]. As confirmed by genomic sequences [20], *N. syl* and *N. tom* are the ancestors of *N. tab*. An interspecific hybridization between *N. syl* and *N. tom* formed *N. Tab.* 0.2 million ago [23]. The three *N. tab* varieties are nearly identical [20]. *N. ben* is a tetraploid (38 chromosomes) that formed ~ 10 million years ago so it is far distinct from tetraploid *N. Tab* (48 chromosomes) [17–21]. Although these genomic sequences are only assembled into contigs and scaffolds, they are sufficiently long in genic regions to allow characterization of SSRs at a whole-genome scale.

Genetic markers have been used in many types of biological research, including the construction of genetic maps, population genotyping, phylogenetics, genome comparisons, gene mapping, quantitative trait loci analysis and marker-assisted breeding [4, 20, 24, 25]. Single nucleotide polymorphisms (SNPs) and SSR markers are the two types of genetic markers most commonly employed nowadays [24]. The most reported markers in *Nicotiana* currently are SSR markers. Two sets of SSR markers, 5119 named PT markers and 4886 named TM or TME markers, were designed from *N. tab* partial genomic DNA or EST sequences by Bindler and colleagues, and by Tong and coworkers, respectively [26, 27]. Of those SSR markers, 2318 PT SSR markers were anchored in 24 linkage groups of a genetic map of tetraploid *N. tab* [27], while 590 SSR markers were placed in another *N. tab* genetic map [26]. In addition, markers were developed and used for the diploid *N. tom* map, involving 489 SSRs and conserved ortholog set II (COS II) markers. The COS II markers are gene sequences of a small number of orthologous loci across the investigated species [28]. Similarly the genetic map of the diploid *N. acuminata* contains a mixture of 308 SSRs and COS II markers [28]. Some of the SSR markers used in the diploid genetic maps are redundant with some of the PT markers.

For the species of *Nicotiana,* all with large genomes, the currently available markers represent only a small part of all possible SSR loci, and these may not be sufficient for some marker-based applications. In addition, most reported SSR markers were developed for one or two particular species or varieties only. Hence, the development of a whole-genome set of SSR markers across multiple species of *Nicotiana* genus is warranted. One complicating issue is that many *Nicotiana* species are polyploid, which can make it difficult to associate any given set of allelic polymorphisms with a particular homoeologous genome. Therefore, the study of markers in multiple genomes of *Nicotiana* will be most helpful if it provides exact allele information. Cross-species marker transferability has also proven to be an important issue during marker development in multiple plant lineages [29]. With the availability of multiple genome sequences of *Nicotiana* species, analysis of both the homoeology and transferability of SSRs markers among species is more feasible, as previously shown [28].

So far, genome-wide SSR distribution has not been well investigated across *Nicotiana* species. Our study discovered and characterized a comprehensive set of SSRs in silico in seven sequenced *Nicotiana* genomes mentioned above. We used our novel software package called GMATA® [7] to facilitate comparative SSR analyses of these large *Nicotiana* genomes, and also developed an efficient protocol for SSR marker development that can be employed across any genome. The discovered *Nicotiana* SSRs and developed SSR markers were used to investigate marker polymorphism in these seven species. We also developed an online database called the Tobacco Markers & Primers Database (TMPD) to facilitate the use of the SSR information and markers.

## Results

### Distributions of SSR types, lengths and locations in *Nicotiana* genomes

We analyzed perfect repeated SSRs with repeat unit length between 1 and 10 bp across seven publicly available *Nicotiana* genome sequences, including three diploids and four tetraploids species. The sequences used in this study total ~ 20 Gb, and are from *N. ben*, *N. syl*, *N. tom*, and *N. oto*, and from *N. tab* cultivars TN90, K326, and BX. These sequences represent a respective ~ 74%, ~ 80%, ~ 78%, ~ 81%, ~ 84%, and ~ 73% of each of the genomes, and are primarily deficient in the highly repetitive regions such as nested transposon blocks that tend to be poor (< 1%) in SSRs [14, 30, 31]. We identified 3010,76 such SSR loci in these seven *Nicotiana* genomes using the software GMATA [7]. The average density of SSRs in the DNA sequence is ~ 155 SSRs/Mb, or one SSR for every ~ 6 kb (Table 1). The properties of SSR occurrence, relative frequency and distribution of each type in these *Nicotiana* genomes were compared in Additional file 1: Table S1.

**Table 1:** Comparison of SSR distribution in seven *Nicotiana* accessions

	*N. ben*	*N. syl*	*N. tom*	*N. oto*	*N. tab* TN90	*N. Tab* K326	*N. Tab* BX	Average
SSR loci	380,475	267,834	230,563	327,525	435,617	430,969	410,049	354,719
Loci distance (kb)	6.8	8.3	7.3	7.6	8.3	8.4	8.7	7.9
Density (SSRs/Mb)	146	120	137	132	120	120	115	127
Genome assembly size (Gb)	2.6	2.2	1.7	2.5	3.6	3.6	3.6	2.8

**Fig. 1:**
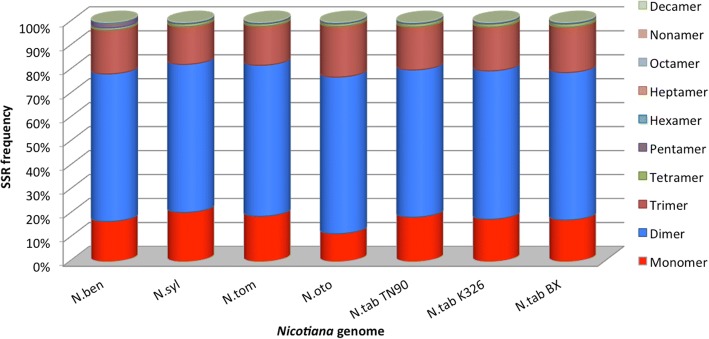
Frequency distributions of different types of SSR repeat units in *Nicotiana* genomes. *N. ben, N. syl, N. tom, N. oto, N. tab* TN90, K326 and BX represent *N. benthamiana*, *N. sylvestris*, *N. tomentosiformis*, *N. otophora*, *N. tabacum* TN90, *N. tabacum* K326, and *N. tabacum* BX, respectively

The types of repeated units in all identified SSRs were characterized. Mono-, di- and tri-nucleotide repeats are the major types of SSRs, accounting for > 98% (Fig. 1). Di-nucleotide repeats are the most frequent SSR type in *Nicotiana*, with a frequency of 61–65% (Fig. 1). Both mono- and tri-nucleotide repeats account for 10–20%, and tetra-nucleotide SSRs comprise about 0.9–1.7% (Fig. 1). The remaining SSR types, from penta-nucleotide to deca-nucleotide, together provide less than 1% of the total SSRs in all investigated *Nicotiana* genomes. However, given these large data sets, this < 1% provides 11,009 long-unit SSR loci that we have discovered in *Nicotiana*. Interestingly, deca-nucleotide SSRs were detected in all investigated *Nicotiana* except *N. tom*. Comparison across seven genomes revealed that six of seven *Nicotiana* genomes have the same abundance rank of di-, mono-, tri- and tetra-nucleotide SSRs while *N. ben* has the order of di-, tri-, mono-, and tetra-nucleotide. The remaining SSR types exhibit different ranks in each species (Additional file 1: Table S1).

SSR polymorphism was then determined by in silico characterization of SSR length variation across the seven *Nicotiana* genomes (Additional file 1: Table S1**)**. SSR length was classified into two categories in accordance with previous designations [32]. The class II type (length < 20 bp) is more abundant than the class I type (length > = 20 bp) in *Nicotiana*. Class II type SSRs account for approximately 70, 68 and 72% of all SSRs in *N. ben*, *N. tom*, *N. oto*, respectively, while there is a higher but very similar percent of ~ 75% in *N. syl* and the three *N. tab* varieties TN90, K326, BX. The similarity of SSR length in the three *N. tab* varieties is consistent with the overall similarity of these genomes [20]. The longer class I SSRs have a greater chance of hyper-polymorphism [32]; therefore, length is an important factor for marker development. The frequency of class I SSRs in *Nicotiana* genomes ranges from 23 to 32%. *N. tom* has a higher percentage (~ 32%) of class I SSRs than does *N. syl* and *N. tab*. There is a similar frequency (~ 25%) of class I SSRs in *N. syl* and *N. tab*. The overall trend of SSR length distribution is that the frequency of occurrence decreases as the SSR length increases (Additional file 2: Figure S1). The rank of SSR abundance in both classes is different between investigated *Nicotiana* varieties and species. *N. ben* and *N. tom* share a very similar abundance order of SSR length when SSR length is < 40 bp, and have species-specific order for SSRs > 40 bp.

**Fig. 2:**
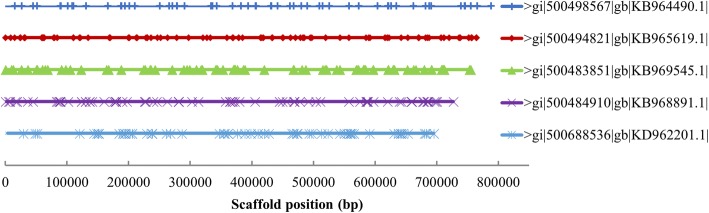
The locations of SSR in *Nicotiana* genome sequences. Plots show SSR locations in five large scaffolds of *Nicotian*a genome sequence. The top five largest sequences were extracted from seven *Nicotiana* genome assemblies, and SSR positions were mined using software GMATA®. The gi number shows the genome sequence ID at GenBank

To investigate the SSR locations in genomic sequences, we extracted the top five longest assemblies (~ 800 kb) from seven genomic assemblies and compared the location of SSRs in these scaffolds. The results showed that the location of SSRs did not follow any obvious pattern in linear genomic sequences (Fig. 2). We further compared the SSR distribution between gene coding sequences (CDS) and all genomic sequences. The results showed that most (99.1%) of the SSR are in non-genic regions, and the longer repeated units of 7–10 bp are represented only in non-genic sequences. The most abundant motif is AT/AT and TA/TA in non-genic sequence while it is TC/GA in CDS (Table 2).

**Table 2:** Comparison of SSR distributions between non-genic sequences and gene coding sequences

	Non-genic sequence	Coding sequence
Sequence (bp) in *N. tabacum* K326	3,598,940,919	97,717,452
Length of repeat unit (bp)	1–10	1–6
Most abundant motif		
AT/TA, TA/AT	37.0%	3.3%
TC/GA, GA/TC	14.90%	34.0%
Total SSRs	524,643 (99.1%)	4660 (0.9%)

### SSR motifs in *Nicotiana*

A detailed characterization of motifs in *Nicotiana* SSRs was carried out using the GMATA software [7]. We grouped the unit motifs into pairs if sequences of two motifs were found to be complementary, because of the unknown orientation of DNA strands, which may lead to different encoded frames, in the current draft genome assemblies. There are 817, 631, 639, 768, 879, 844, and 847 types of grouped motif pairs identified in *N. ben*, *N. syl*, *N. tom*, *N. oto*, and *N. tab* TN90, K326, BX, respectively. Generally, the abundance of grouped motif pairs in *Nicotiana* increased with the size of the genome sequence. *N. tab* has the highest number of grouped motif pairs, but this number differs somewhat between *N. tab* varieties. The motif pairs ranking at the 20 highest frequencies in each of the seven *Nicotiana* genomes are shown in Additional file 1: Table S1. Motif pairs AT/AT are predominant, accounting for 44–50% of total SSRs in the seven examined *Nicotiana* genomes. Grouped motif pairs GC/GC and CG/CG are the rarest di-nucleotide motifs, at frequencies of ~ 0.1–0.17%, ranking after twenty tri-nucleotide motif pairs. The distribution of tri-nucleotide motif pairs displayed major genetic variation among these *Nicotiana* genomes. Motif pairs CAA/TTG and GTT/AAC are the most abundant tri-nucleotides, with a total frequency of ~ 5% in six of the genomes, but *N. tom* has more motif pair ATT/AAT than motif pair CAA/TTG and GTT/AAC. AAAT/ATTT is the most abundant tetra-nucleotide motif pair in six of the genomes, but is the second most abundant in *N. ben,* after the ATAC/GTAT motif pair. The remaining motif pairs with repeat lengths > 4 bp are the rarest motifs (Additional file 1: Table S1).

### Genome-wide SSR marker development in multiple *Nicotiana* genomes

To develop a comprehensive set of SSR markers at a genomic sequence scale in multiple *Nicotiana* genomes, the sequences flanking SSR loci in the seven *Nicotiana* genomic sequences were used to design PCR primers with the GMATA software [7]. In total, 1,943,197 loci (78.3% of the total analyzed) yielded high quality primer pairs. Of the seven *Nicotiana* genomes, most genomic sequences are highly similar [18, 20]; therefore, it is likely that designed primers for one genome will commonly work in another. In our approach, the same primer pairs designed from more than one genome dataset were condensed to a single unique primer pair. Further clustering was conducted to keep unique primer pairs based on identity of the primer’s sequence. Each unique SSR primer pair was then considered as a potential marker and assigned a unique marker ID. In total, 1,224,048 unique SSR markers were designed. We call these NIX (*Nicotiana* multiple (X) genome) markers. The information on all designed NIX markers is provided in “sts” format, according to the NCBI’s rule for marker information, and is freely available to the public in our online database TMPD as described later.

To investigate the novelty of the NIX markers, a comparison was conducted between NIX markers and previously published markers in *Nicotiana*. After removing redundancy of published markers based on primer sequence similarity, 5397 unique SSR markers from Bindler et al. [27, 33] and 387 unique markers from Wu et al. [28, 34] were classified as reported markers. Of these, 2143 (37.0%) of the reported markers exhibited only one primer that overlapped with those in NIX markers. A total of 264 of the NIX markers yielded overlapping primers for both members of a pair, and are thus identified as duplications of published marker pairs, indicating that 99.98% of NIX makers are novel (Additional file 2: Figure S1). However, a few novel NIX markers do amplify the same SSR loci published in earlier studies [27, 28, 33, 34].

### In silico analysis of NIX marker polymorphism across genomes

**Fig. 3:**
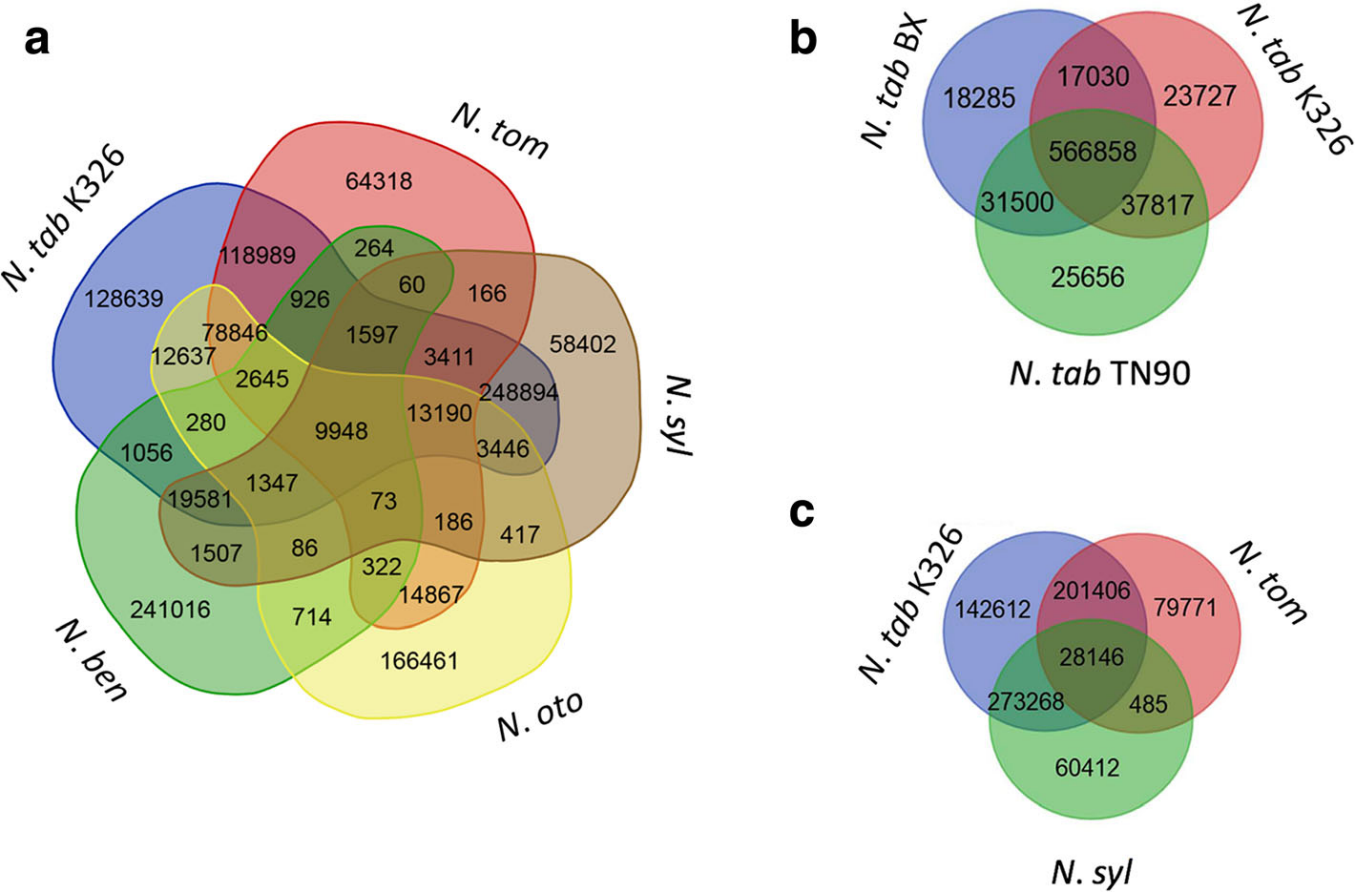
Venn diagram showing the number of common and specific NIX markers in *Nicotiana* species and varieties (**a**), varieties of *N. tab* (**b**), *N. tab* K326 and putative progenitors (**c**). *N. ben, N. syl, N. tom, N. oto, N. tab* TN90, K326 and BX represent *N. benthamiana*, *N. sylvestris*, *N. tomentosiformis*, *N. otophora*, *N. tabacum* TN90, *N. tabacum* K326, and *N. tabacum* BX, respectively

For marker utilization, it is vital to know the amplification efficiency, allele size, and level of polymorphism (i.e., number of alleles). Thus, an in silico simulated PCR was carried out to investigate polymorphism across the seven *Nicotiana* genomic sequences. Two copies of genes in tetraploid *Nicotiana* will produce two alleles of PCR product if repeat numbers are different. We found that all the designed NIX markers would produce at least one amplicon in at least one investigated genome. In the diploids, ~ 30% of designed NIX markers would amplify DNA in *N. syl* while a lower percentage (~ 25%) would provide an amplification product in both *N. tom* and *N. oto*. For tetraploids, ~ 53% of NIX markers are amplifiable in each of the three *N. tab* varieties, while only ~ 23% would amplify an SSR product in *N. ben*. Because the information regarding common and species-specific NIX markers will be extremely useful for marker selection during application, we checked this for all NIX markers. A total of 9948 NIX markers (~ 0.8%) will be amplifiable in all seven investigated *Nicotiana* species or varieties, so we infer these markers are very likely to be amplifiable in any other *Nicotiana* species. There are also many species-specific NIX markers (e.g., 128,639 such markers in *N. tab*). *N. ben* has more species-specific NIX markers than any other, which may be explained by the fact that *N. ben* is the most distantly related of the *Nicotiana* species analyzed [19]. The fewest NIX markers were found in *N. syl* (Fig. 3a). Of the NIX markers in the three *N. tab* varieties, 566,858 (78.6%) are in common, while only a few markers (2.3–3.5%) are variety-specific (Fig. 3b). Despite the low divergence between the *N. tab* varieties, only ~ 3.6% (28,416) of the NIX markers were shared with both progenitors *N. syl* and *N. tom*. Besides the common NIX markers, *N. tab* appears to have inherited a different number of NIX markers from its two putative progenitors, 34.8% from *N. syl* and 25.6% from *N. tom* (Fig. 3c).

A total of 744,993 (61%), 211,845 (17%) and 106,472 (8.7%) of the designed NIX markers are predicted to produce a respective one, two or three PCR fragments from the combined sequences of these seven genomes. Some NIX markers will amplify only from one or a few *Nicotiana* genomes. Of the amplifiable NIX markers in a given genome, on average, ~ 81% of the NIX markers are predicted to produce one fragment while ~ 8% will produce two fragments. Species *N. ben* has the lowest percent (~ 6%) of NIX markers producing two fragments while *N. oto* has the highest percent (~ 11%). In the seven *Nicotiana* genomes combined, ~ 3.8% of NIX markers yield many predicted fragments (> = 11), suggesting that they may be amplified from repeated DNA sequences.

For all 1,224,048 NIX markers, the predicted amplicons were scored for predicted length polymorphisms across the seven genomic sequences (Table 3). A total of 430,848 markers (35.2%) showed polymorphism between at least two of the investigated *Nicotiana* genomes. The polymorphism information is available in the TMPD database.

**Table 3:** A portion of the table showing amplification information for NIX markers in the seven *Nicotiana* genomes

Marker_ID	*N. ben*	*N. syl*	*N. tom*	*N. oto*	*N. tab* TN90	*N. Tab* K326	*N. Tab* BX	Polymorphic	Allele #
>NIX189	NA	347	NA	NA	347	347	347	No	1
>NIX190	697	232	NA	NA	234	234	234	Yes	3
>NIX191	NA	NA	NA	NA	179	179	179	No	1
>NIX192	NA	285	NA	NA	287	287	287	Yes	2
>NIX193	155	157	155	157	155 + 157	155 + 157	155 + 157	Yes	2
>NIX194	NA	816	NA	NA	NA	233	NA	Yes	2
>NIX195	NA	NA	NA	NA	202	215	201	Yes	3
>NIX196	NA	208	NA	NA	207	207	207	Yes	2
>NIX197	NA	286	NA	NA	286	286 + 287	286	Yes	2
>NIX198	NA	128 + 278 + 308 + 338	NA	NA	128	128	128	No	1
>NIX199	NA	278 + 308 + 338	NA	NA	278 + 308	278 + 308	278 + 308	Yes	3

Each marker has a unique ID, starting with the prefix >NIX. Data indicates the amplicon’s length (bp) in the format of in silico predicted size of the PCR fragment(s) in each *Nicotiana* germplasm. NA indicates that the amplicon should not be found in this genome. *N. ben*, *N. syl, N. tom, N. oto, N. tab* TN90*, N. Tab* K326*, and N. Tab* BX*,* represent *N. benthamiana, N. sylvestris, N. tomentosiformis*, *N. otophora*, and *N. tabacum* TN90, K326, and BX, respectively

### Experimental validation of SSR markers

**Fig. 4:**
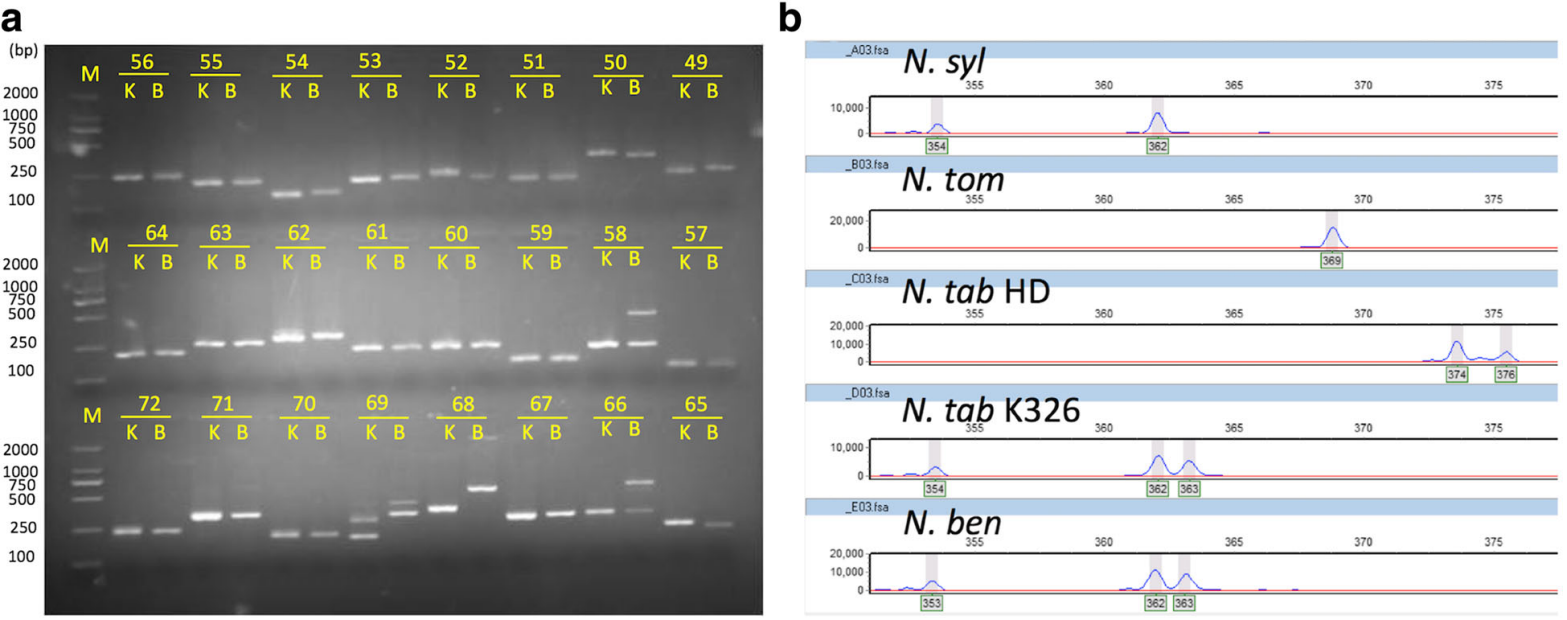
Experimental validations of NIX markers by amplification and allele scoring. Image shows the PCR fragments resolved by agarose gel (**a**) or DNA fragment analyzer ABI3730X (**b**). *N. syl, N. tom, N. Tab HD, N. Tab K326,* and *N. ben* represent *N. sylvestris, N. tomentosiformis*, *N. tabacum* HD, *N. tabacum* K326 (K), and *N. benthamiana* (B)*,* respectively. The numbers in the image represent validated IDs for markers

In total, 120 NIX markers were randomly selected from the predicted commonly amplifiable markers for experimental validation by PCR amplification on a testing panel of five *Nicotiana* accessions, including four *Nicotiana* species of *N. syl, N. tom. N. Tab* K326 and *N. ben* with known genomic sequence and additional species *N. glutinosa* or accession *N. Tab* HD with unknown genomic sequence (Additional file 3: Table S2). Of the tested NIX markers, 100% produced amplicons from at least four of the entire panel (Fig. 4a, Additional file 3: Table S2). For each of the *Nicotiana* accession, more than 99% of the markers produced amplicons in the expected size range. High amplification rates of these markers in the species *N. glutinosa* or accession *N. Tab* HD may indicate a high level of usefulness in other *Nicotiana* species or accessions. For most of the tested NIX markers, it is difficult to score the polymorphism among accessions in an agarose gel; however, most of the predicted polymorphisms were easily identified by fluorescent genotyping using the ABI3730X DNA fragment analyzer (Fig. 4b). We further tested the polymorphism of 24 markers with an ABI3730X, and found a high resolution of polymorphic bands (Fig. 4b). The validation results and gel images are available in our online database, TMPD, described later. The validation results demonstrated that *Nicotiana* NIX markers will be valuable tools that are applicable for multiple *Nicotiana* species or varieties.

### Online database of tobacco SSR markers

**Fig. 5:**
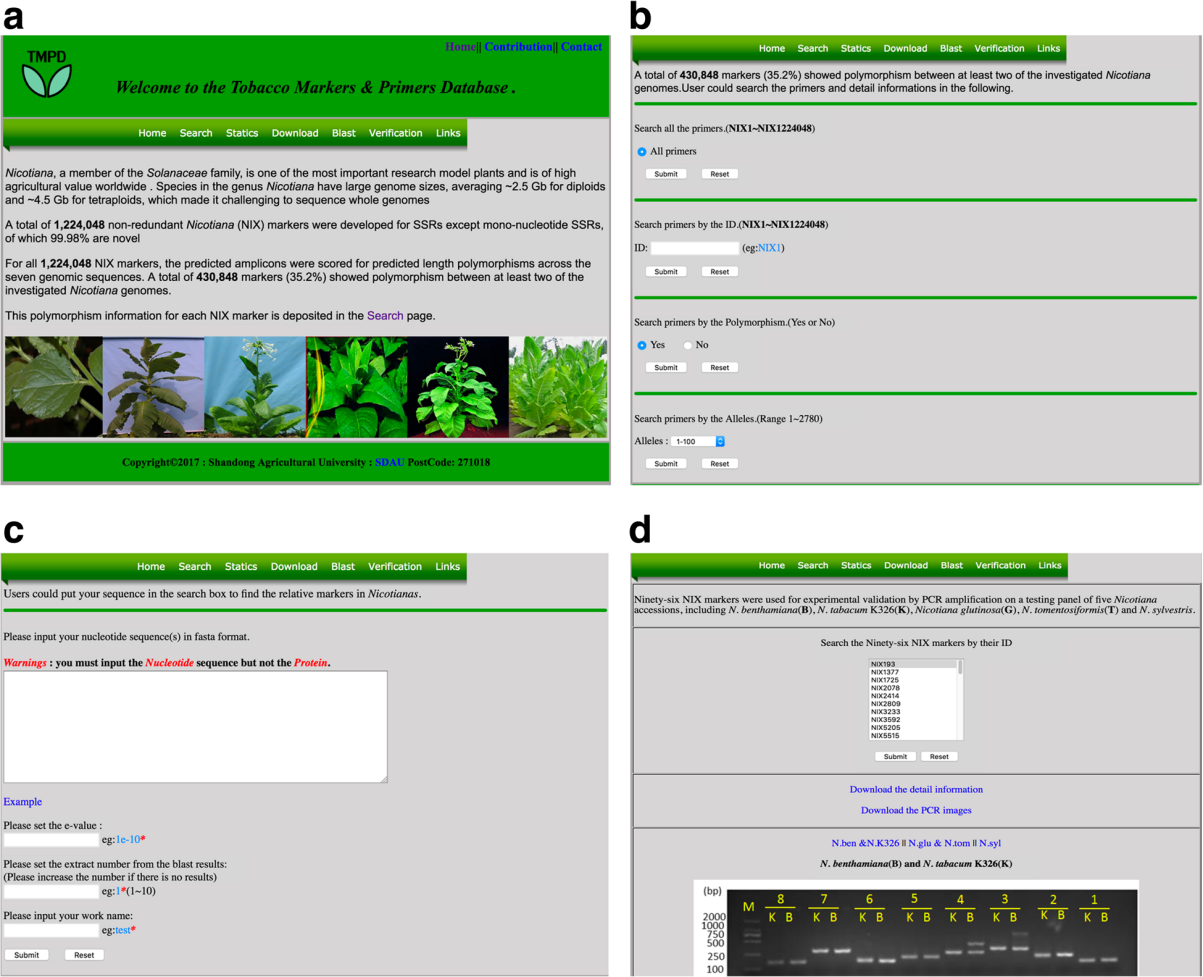
The online tobacco markers and primers database TMPD. The TMPD database is freely available online at http://biodb.sdau.edu.cn/tmpd/index.html. Images **a, b, c**, and **d** show the TMPD interface, search function, Blast function and validation search function

To facilitate the application of NIX markers, we constructed a freely available online database called the Tobacco Markers & Primers Database (TMPD) (http://biodb.sdau.edu.cn/tmpd/index.html, Fig. 5a). All NIX marker information, including primer sequences, fragment sizes, polymorphism levels and genomic location, can be downloaded. Searches can be done by NIX ID number, by primer sequence or by DNA sequence using our online Blast function (Fig. 5b and c). If the user has a DNA sequence, the Blast function in TMPD can find the nearest NIX markers to the sequence, and a further click will show the NIX marker information. A function for archiving and searching validated results was built into TMPD. TMPD hosts our existing marker validation results including gel images, and fragment information (Fig. 5d). In addition, TMPD can accept new validation results from any users.

## Discussion

SSRs have been comprehensively characterized for some plant species after their genome sequences were published, including in *Brachypodium* [10], foxtail millet [12, 35, 36], *Brassica* [13], and cassava [37]. Currently, SSR DNA entries from 110 plant species with sequenced genomes are available in a plant microsatellite DNAs database (PMDBase) [38]. However, the genome-scale distribution of SSRs in the *Nicotiana* genus has been lacking due to the absence of a whole genome sequence. Recently, the availability of seven extensive genome sequence analyses from the *Nicotiana* genus have made deep investigation of SSRs possible [17]. The genomes, providing ~ 20 Gb of sequence data, include information from diploids and tetraploids that are closely related to commercial tetraploid tobacco, *N. tab*. This is the first report of the comprehensive identification, distribution analysis and comparison of SSRs in seven deeply sequenced genomes from the genus *Nicotiana*. To efficiently discover SSRs in large scale genome sequence data sets, the recently published software package GMATA® [7] was used. We discovered that mono-, di- and tri-nucleotide types of all the repeat units with lengths ranging from 1 bp to 10 bp are the most abundant. This observation in *Nicotiana* is consistent with the typical features of SSR distribution in dicots revealed by a comparison of 29 plant species [6] and in *Brassica* [5]. Grouped motifs TA/TA and AT/AT are predominant (44–50%), while GC/GC and CG/CG are the rarest di-nucleotides in all investigated *Nicotiana* genomes. In *Nicotiana*, most (> 68%) of the SSRs are short (< 20 bp), which is routinely correlated with a relatively low level of polymorphism for these markers. Our comparison of distribution revealed that SSRs in *Nicotiana* are mainly located in the regions between genes. This observation may partly explain the low rate (~ 4%) of polymorphism identified during *Nicotiana* SSR marker screening in an earlier study [39] that used genic sequences as the starting point for SSR discovery.

Previously, studies on *Nicotiana* SSRs were focused on a specific genome or variety. In this study, we compared SSRs between tetraploid *N. tab* and its ancestral diploids, between varieties of tetraploid *N. tab*, and between different tetraploid species. Comprehensive comparison revealed genome-specific SSRs in each of the seven *Nicotiana* genomes. Some of the absences of SSRs in certain accessions may result from incompleteness in the genome sequences. However, most genomic sequences that are missing in these seven assemblies are repetitive sequences that are generally deficient in SSRs [30]. Therefore, the SSRs represented here should reflect the general distribution of SSRs. Species *N. Tab* BX and *N. ben*, for instance, have incomplete genome sequences, believed to represent 74 and 73% of full genome sequence, respectively [19, 20]. However, *N. Tab* BX did not show a biased distribution of amplicons by in silico analysis compared with the other genomes. *N. ben* has the fewest NIX markers that are predicted in silico to produce two fragments, which may be caused by both the less complete sequences and perhaps by the fewer chromosomes (38) in this species, compared to the 48 chromosomes in other tetraploids. Perhaps the chromosome number reduction was associated with some DNA loss during tetraploid evolution.

Diploid *N. oto* has more grouped motifs than diploid *N. syl*, which indicates that the two genomes became very different after their lineages diverged from the same ancestor. This conclusion is also supported by genome sequence comparison of these two species [20]. In tetraploids, the three varieties of *N. tab* have very similar numbers of grouped motifs, indicating their limited degree of divergence. This high homology, also seen in earlier studies [20], is likely to reflect both recent common ancestry and human selection for desirable traits linked to some of these NIX markers. Regarding the types of SSRs, all investigated *Nicotiana* genomes have similar di-nucleotide type abundances. However, mono-nucleotide, tri-nucleotide and other higher-order repeats were found to vary a great deal among *Nicotiana* genomes. These long SSRs are the major differences in SSRs between these *Nicotiana* genomes. In bacteria, previous studies have shown that many SSRs in intergenic or upstream regions can have regulating functions [6], and this function may sometimes be true in large-genome eukaryotes. It will be interesting to investigate whether any of these differences will lead to phenotypic variation, particularly for SSRs within transcribed regions of genes or in regulatory domains of the adjacent chromatin. Regarding SSR length, *N. ben* and *N. tom* shared a very similar abundance order. The similar distribution of short SSR repeats in *N. syl* and *N. tab* may be partially explained by the fact that *N. tab* has retained more genomic sequence from *N. syl* than from *N. tom* [20, 22, 23]. We found that longer SSRs (> 40 bp) are usually genome-specific in *Nicotiana*. In conclusion, all genomes shared most of their SSR motifs while each genome has its own specific motifs, especially for larger repeat lengths and distribution frequencies.

SSR evolution can be revealed by comparison of the SSR and its flanking sequence. The NIX markers were designed from the sequences flanking SSRs, so the amplicons’ lengths in different *Nicotiana* genomes will provide insights into SSR evolution. SSRs in allopolyploids of *N. tab* showed considerable departure from the predicted additivity of their ancestral progenitors, *N. syl* and *N. tom*, which was demonstrated by the distribution of shared and specific NIX markers (Fig. 3c). There is an increased number of amplifiable NIX markers (142,612) in *N. tab* compared to the sum of *N. syl*-specific NIX marker (60,412) and *N. tom*-specific NIX markers (79,771). This difference may partially be caused by both the incomplete genome sequences and real biological differences. An increase in amplifiable NIX markers (net increase of 2429 or 1.7%) means increased SSRs in polyploids relative to their diploid progenitors. In contrast, SSR frequencies in *Brassica* species were observed to decrease after polyploidization [5]. The genome size in *N. tab* is less than the sum of their ancestral progenitors, *N. syl* and *N. tom* (Leitch et al. 2008). It has been previously noted that the genome reduction from the *N. tom* ancestor was greater than that from the maternal *N. syl* genome in natural *N. tab* [20, 22, 23]. This suggests that the extra SSRs in *N. tab* may be from the amplification of DNA from the *N. syl*-like ancestor and loss of DNA from *N. tom* ancestor, which is also supported by the genomic sequence comparison between species [20]. In addition, the variety-specific and species-specific NIX markers in the polyploids indicate either SSR formation or loss after the polyploidization or speciation events. Alternatively, this may reflect differential transmission of SSR polymorphisms from the ancestors.

SSR markers have been used in several genetic maps of *Nicotiana* species. For tetraploid *N. tab*, the genetic map contains around 2000 SSR markers [27, 33]. In the diploid *N. acuminata* and *N. tom* genetic maps, a mixture of 489 and 308 SSR plus COSII markers were also reported, respectively [28]. The total number of NIX markers designed here were more than one million, 99.98% of which do not overlap with previous markers in *Nicotiana*. Thus, the NIX markers reported here have increased, about 240-fold, the SSR marker resource for the *Nicotiana* community. We identified 9948 SSR markers that were informative on all seven *Nicotiana* investigated. These transferable SSRs will facilitate future characterization of genomic diversity, relatedness and evolution across the entire *Nicotiana* genus.

The great value of SSR markers is their co-dominance and allelic multiplicity. The information for predicted allele number and size were determined and provided for each NIX marker in each of the seven genomes, so this will be an immediately useful resource for marker-assisted selection and genetic studies. This is the first report of comparative marker amplification information across multiple genomes of the same genus, and our TMPD database makes it easy to search for and compare the information for each marker, which will facilitate the efficiency of NIX markers use to follow linked agronomic or other genetic traits in future studies. In total, ~ 94% of NIX markers amplify 1–2 alleles per genome. The remaining NIX markers may be less useful for application due to multiple copies in a single genome, which is a possible outcome of their presence in repeated sequences such as transposable elements. In the past decade, molecular breeding using DNA markers has become the norm with many crop species [40–42]. The informative NIX markers developed here can be effectively used in genotyping of populations, QTL identification of interesting traits, germplasm diversity analysis, and marker-assisted selection in *Nicotiana*.

A highly efficient experimental protocol for the use of SSR markers was developed here, as well. Because all NIX markers were designed for uniform PCR conditions, the PCR reaction can be conducted at the single annealing temperature of 60 °C. For the standard PCR setting, the user will just add DNA, primers, and water to our standard master mix. This will facilitate rapid PCR application. Our PCR-based experimental validation of 120 NIX markers across five species or varieties demonstrated that the method worked in 100% of the cases investigated. Thus, the PCR product scoring method for NIX is of high efficiency, high resolution and high throughput with little background noise. Unlike the broad/fuzzy peaks seen with much PCR/SSR analysis, the sharp peaks in our method may result from the fully functional Taq polymerase employed, which retains the 3′-5′ exonuclease activity. In addition, our PCR settings for NIX marker have a high stringency that leads to the exclusive production of specific PCR fragments.

## Conclusions

We characterized SSR in seven genomes of *Nicotiana* genus, developed NIX markers for these genomes, and constructed an online database for using the information. The NIX markers greatly expand *Nicotiana* marker resources, thus providing an immediately useful tool for research and breeding. We demonstrated a novel protocol for SSR marker development and SSR utilization at the whole genome scale that can be applied to any lineage of organisms. We also developed the TMPD database to facilitate NIX marker utility.

## Methods

### *Nicotiana* genomic sequence resources

The genomic sequences of seven *Nicotiana* used here are from publicly available databases. The draft assembly (version 0.44) of the genome sequence for *N. ben* was retrieved from the web site of Solgenomics. The genome sequences of *N. syl* (assembly ID: GCA_000393655.1, NCBI Accession No. ASAF00000000), *N. tom* (assembly ID: GCA_000390325.1, Accession No. ASAG00000000), *N. oto* (Accession No. AWOL000000000.1), *N. tab* TN90 (Accession No. AYMY00000000.1), *N. Tab* K326 (Accession No. AWOJ00000000.1) and *N. tab* Basma Xanthi (Accession No. AWOK00000000.1) were retrieved from Genbank at NCBI using the web links as described previously [17].

### SSR discovery and analysis

Perfect SSRs, consisting of perfect repetitions of the same kind of nucleotide unit, were mined from whole genomic sequences of *Nicotiana* species by using our published software package, GMATA [7] (http://sourceforge.net/projects/gmata/). The parameters of SSR unit length were set to at least 12 bp for mono-nucleotide repeats, and at least five tandem copies for 2 bp to 10 bp nucleotide motifs. The five tandem copies criterion was used from our previous experiments [43] and other recent publications [44, 45].

### Primer and marker design

Primers were designed in the sequence regions flanking each SSR locus by using the GMATA software package [7]. These settings were: product size ranging from 120 to 400 bp, targeted Tm of 60 °C (57–62 °C), primer target length of 20 nt (18–25 nt), minimum GC of 40%, maximum GC of 65% and optimized GC at 50%, allowed maximum poly-X 4, maximum self-complementarity 6, maximum 3′ self-complementarity 2, and maximum Ns 1. The size range of PCR products was chosen to permit easy fragment scoring in gels and DNA fragment size analyzers such as the ABI3730X.

SSR markers were then computed from the potential primer pairs. All designed primers were investigated for sequence identity. If sequences of both forward and reverse primer were 100% identical to those of other primer pairs, the primer pair was considered as a redundant primer pair. Only unique primer pairs were kept. An SSR marker was then recorded after assigning a unique sequential marker ID with the NIX prefix for each unique primer pair.

### In silico marker mapping to genomes

To map the designed marker to genomic sequences, the GMATA software package [7] was used to perform an in silico amplification by calling the e-PCR algorithm [46]. The e-PCR result was used to process the marker mapping information. The settings for e-PCR were margin 3000, no gap in primer sequence, no mismatch in primer sequence, allowed size range of 100–1000, word size (−w) 12 and contiguous word (−f) 1. The mapping information in the results includes mapped or unmapped markers, the alleles of each marker, and marker anchoring information in each sequence of a genome. All designed markers were used to map to sequences of each *Nicotiana* genome, in silico*,* separately.

### Experimental PCR validation of SSR markers

To test whether the newly-designed SSR markers were amplifiable, experimental PCR was conducted in several *Nicotiana* species and varieties. Genomic DNA was extracted from leaves of *N. ben*, *N. syl*, *N. tom*, *N. tab* variety K326, and *N. tab* variety HD or *N. glutinosa* by using the DNeasy Plant Mini Kit (Qiagen, Cat. No. 69104). DNA was diluted to 30–50 ng/μl in water before use. 120 markers were randomly chosen from the predicted commonly amplifiable makers across seven *Nicotiana* genomes for validation by using PCR amplification (Additional file 3: Table S2). For 96 markers scored in agarose gel only, PCR was performed in 20 μl reaction volumes containing 30–50 ng (1 μl) DNA, 0.53 μM tailed forward primer, 0.53 μM reverse primer, and 10 μl Phusion Hot Start Flex 2X Master Mix (NEB, Cat. No. M0536S). For 24 markers used in further fragment scoring, a tailed forward primer was synthesized with tail sequence CGTTGTAAAACGACGGCCAGT added to the 5′ end of each marker’s forward primer. PCR was performed in 20 μl volumes containing 30–50 ng (1 μl) DNA, 0.13 μM tailed forward primer, 0.27 μM 5’ FAM or 5’ HEX florescent labeled primer CGTTGTAAAACGACGGCCAGT, 0.53 μM reverse primer, and 10 μl Phusion Hot Start Flex 2X Master Mix (NEB, Cat. No. M0536S). PCR amplification and amplicon scoring were conducted according to our previous description for UGSW markers [43]. 15 μl of remaining amplification product were resolved in a 2.5% agarose gel. The PCR product from 24 markers with tail were further scored in ABI3730X sequence for high resolution fragment screening.

### Comparison between reported markers and NIX markers

All publicly available *Nicotiana* markers were downloaded from the websites. These data included all amplifiable *N. tab* SSR markers reported previously [27, 33], and a mixture of 489 and 308 SSR and COSII markers from *N. acuminata* and *N. tom* genetic maps, respectively [28]. Redundant markers were sought and removed based on sequence identity of both primers for all markers after merging into one data set. From this analysis, we obtained 5397 SSR markers and 387 SSR or COSII markers from previous studies. A BLASTN search against reported non-redundant marker sequences was conducted to investigate the novelty of NIX markers with BLASTN algorithm in BLAST+ software and the settings are at -m8 –b5 -v5 –e 1e-5 and at least 95% identity [47]. NIX markers were identified as redundant markers if one or more primer sequence matched with those of reported markers.

## Additional files

Additional file 1: **Table S1** Genomic SSR distribution of *Nicotiana. (XLS 469 kb)*

Additional file 2: **Figure S1** SSR length distribution. (PDF 364 kb)

Additional file 3: **Table S2** Duplication between NIX and reported markers. (XLS 41 kb)

Additional file 4: **Table S3** Information for experimentally validated markers. (XLS 51 kb)

## Abbreviations

BX: Basma Xanthi
*N. ben*: *N. benthamiana*
*N. oto*: *N. otophora*
*N. syl*: *N. sylvestris*
*N. Tab*: *N. tabacum*
*N. tom*: *N. tomentosiformis*
NIX: *N**i**cotiana* multiple (X) genome markers
SSR: Simple sequence repeat
TMPD: Tobacco markers & primers database

## References

[1] Ellegren H (2004). Microsatellites: simple sequences with complex evolution. Nat Rev Genet.

[2] Klintschar M, Dauber E-M, Ricci U, Cerri N, Immel U-D, Kleiber M, Mayr WR (2004). Haplotype studies support slippage as the mechanism of germline mutations in short tandem repeats. Electrophoresis.

[3] Forster P, Hohoff C, Dunkelmann B, Schürenkamp M, Pfeiffer H, Neuhuber F, Brinkmann B (1803). Elevated germline mutation rate in teenage fathers. Proc R Soc Lond [Biol].

[4] Kumar M, Choi JY, Kumari N, Pareek A, Kim SR. Molecular breeding in *Brassica* for salt tolerance: importance of microsatellite (SSR) markers for molecular breeding in *Brassica*. Front Plant Sci. 2015;6:688. 10.3389/fpls.2015.00688 PMC4559640 26388887

[5] Shi J, Huang S, Fu D, Yu J, Wang X, Hua W, Liu S, Liu G, Wang H (2013). Evolutionary dynamics of microsatellite distribution in plants: insight from the comparison of sequenced *Brassica*, *Arabidopsis* and other angiosperm species. PLoS One.

[6] Zhao Z, Guo C, Sutharzan S, Li P, Echt CS, Zhang J, Liang C (2014). Genome-wide analysis of tandem repeats in plants and green algae. G3.

[7] Wang X, Wang L (2016). GMATA: an integrated software package for genome-scale SSR mining, marker development and viewing. Front Plant Sci.

[8] Zhang Z, Deng Y, Tan J, Hu S, Yu J, Xue Q (2007). A genome-wide microsatellite polymorphism database for the *Indica* and *Japonica* rice. DNA Res.

[9] Song Q, Jia G, Zhu Y, Grant D, Nelson RT, Hwang E-Y, Hyten DL, Cregan PB (2010). Abundance of SSR motifs and development of candidate polymorphic SSR markers (BARCSOYSSR_1.0) in soybean. Crop Sci.

[10] Sonah H, Deshmukh RK, Sharma A, Singh VP, Gupta DK, Gacche RN, Rana JC, Singh NK, Sharma TR (2011). Genome-wide distribution and organization of microsatellites in plants: an insight into marker development in *Brachypodium*. PLoS One.

[11] Xu J, Liu L, Xu Y, Chen C, Rong T, Ali F, Zhou S, Wu F, Liu Y, Wang J (2013). Development and characterization of simple sequence repeat markers providing genome-wide coverage and high resolution in maize. DNA Res.

[12] Pandey G, Misra G, Kumari K, Gupta S, Parida SK, Chattopadhyay D, Prasad M (2013). Genome-wide development and use of microsatellite markers for large-scale genotyping applications in foxtail millet *Setaria italica* (L.). DNA Res.

[13] Shi J, Huang S, Zhan J, Yu J, Wang X, Hua W, Liu S, Liu G, Wang H (2014). Genome-wide microsatellite characterization and marker development in the sequenced *Brassica* crop species. DNA Res.

[14] Wang Q, Fang L, Chen J, Hu Y, Si Z, Wang S, Chang L, Guo W, Zhang T (2015). Genome-wide mining, characterization, and development of microsatellite markers in *Gossypium* species. Sci Rep.

[15] Ow DW, De Wet JR, Helinski DR, Howell SH, Wood KV, Deluca M (1986). Transient and stable expression of the firefly *Luciferase* gene in plant cells and transgenic plants. Science.

[16] Senthil-Kumar M, Mysore KS (2011). New dimensions for VIGS in plant functional genomics. Trends Plant Sci.

[17] Wang X, Bennetzen JL (2015). Current status and prospects for the study of *Nicotiana* genomics, genetics, and nicotine biosynthesis genes. Mol Gen Genomics.

[18] Sierro N, Battey J, Ouadi S, Bovet L, Goepfert S, Bakaher N, Peitsch M, Ivanov N (2013). Reference genomes and transcriptomes of *Nicotiana sylvestris* and *Nicotiana tomentosiformis*. Genome Biol.

[19] Bombarely A, Rosli HG, Vrebalov J, Moffett P, Mueller L, Martin G (2012). A draft genome sequence of *Nicotiana benthamiana* to enhance molecular plant-microbe biology research. Mol Plant-Microbe Interact.

[20] Sierro N, Battey JND, Ouadi S, Bakaher N, Bovet L, Willig A, Goepfert S, Peitsch MC, Ivanov NV. The tobacco genome sequence and its comparison with those of tomato and potato. Nat Commun. 2014;5:3833. 10.1038/ncomms4833 PMC4024737 24807620

[21] Sierro N, van Oeveren J, van Eijk MJT, Martin F, Stormo KE, Peitsch MC, Ivanov NV (2013). Whole genome profiling physical map and ancestral annotation of tobacco Hicks broadleaf. Plant J.

[22] Renny-Byfield S, Chester M, Kovařík A, Le Comber SC, Grandbastien MA, Deloger M, Nichols RA, Macas J, Novák P, Chase MW (2011). Next generation sequencing reveals genome downsizing in allotetraploid *Nicotiana tabacum*, predominantly through the elimination of paternally derived repetitive DNAs. Mol Biol Evol.

[23] Leitch IJ, Hanson L, Lim KY, Kovarik A, Chase MW, Clarkson JJ, Leitch AR (2008). The ups and downs of genome size evolution in polyploid species of *Nicotiana* (Solanaceae). Ann Bot.

[24] Davey JW, Hohenlohe PA, Etter PD, Boone JQ, Catchen JM, Blaxter ML (2011). Genome-wide genetic marker discovery and genotyping using next-generation sequencing. Nat Rev Genet.

[25] Mauro-Herrera M, Wang X, Barbier H, Brutnell TP, Devos KM, Doust AN (2013). Genetic control and comparative genomic analysis of flowering time in *Setaria* (Poaceae). G3.

[26] Tong Z, Yang Z, Chen X, Jiao F, Li X, Wu X, Gao Y, Xiao B, Wu W (2012). Large-scale development of microsatellite markers in *Nicotiana tabacum* and construction of a genetic map of flue-cured tobacco. Plant Breed.

[27] Bindler G, Plieske J, Bakaher N, Gunduz I, Ivanov N, Van der Hoeven R, Ganal M, Donini P (2011). A high density genetic map of tobacco (*Nicotiana tabacum* L.) obtained from large scale microsatellite marker development. Theor Appl Genet.

[28] Wu F, Eannetta N, Xu Y, Plieske J, Ganal M, Pozzi C, Bakaher N, Tanksley S (2010). COSII genetic maps of two diploid *Nicotiana* species provide a detailed picture of synteny with tomato and insights into chromosome evolution in tetraploid *N. tabacum*. Theor Appl Genet.

[29] Moon HS, Nicholson JS, Lewis RS (2008). Use of transferable *Nicotiana tabacum* L. microsatellite markers for investigating genetic diversity in the genus *Nicotiana*. Genome.

[30] Morgante M, Hanafey M, Powell W (2002). Microsatellites are preferentially associated with nonrepetitive DNA in plant genomes. Nat Genet.

[31] SanMiguel P, Tikhonov A, Jin Y-K, Motchoulskaia N, Zakharov D, Melake-Berhan A, Springer PS, Edwards KJ, Lee M, Avramova Z (1996). Nested retrotransposons in the intergenic regions of the maize genome. Science.

[32] Temnykh S, DeClerck G, Lukashova A, Lipovich L, Cartinhour S, McCouch S (2001). Computational and experimental analysis of microsatellites in rice (*Oryza sativa* L.): frequency, length variation, transposon associations, and genetic marker potential. Genome Res.

[33] Bindler G, van der Hoeven R, Gunduz I, Plieske J, Ganal M, Rossi L, Gadani F, Donini P (2007). A microsatellite marker based linkage map of tobacco. Theor Appl Genet.

[34] Wu F, Eannetta N, Xu Y, Durrett R, Mazourek M, Jahn M, Tanksley S (2009). A COSII genetic map of the pepper genome provides a detailed picture of synteny with tomato and new insights into recent chromosome evolution in the genus *Capsicum*. Theor Appl Genet.

[35] Zhang S, Tang C, Zhao Q, Li J, Yang L, Qie L, Fan X, Li L, Zhang N, Zhao M (2014). Development of highly polymorphic simple sequence repeat markers using genome-wide microsatellite variant analysis in foxtail millet *Setaria italica* (L.) P. Beauv. BMC Genomics.

[36] Kumari K, Muthamilarasan M, Misra G, Gupta S, Subramanian A, Parida SK, Chattopadhyay D, Prasad M (2013). Development of eSSR-markers in *Setaria italica* and their applicability in studying genetic diversity, cross-transferability and comparative mapping in millet and non-millet species. PLoS One.

[37] Vasquez A, Lopez C (2014). *In silico* genome comparison and distribution analysis of simple sequences repeats in cassava. Int J Genomics.

[38] Yu J, Dossa K, Wang L, Zhang Y, Wei X, Liao B, Zhang X (2017). PMDBase: a database for studying microsatellite DNA and marker development in plants. Nucleic Acids Res.

[39] Tong Z, Jiao T, Wang F, Li M, Leng X, Gao Y, Li Y, Xiao B, Wu W (2012). Mapping of quantitative trait loci conferring resistance to brown spot in flue-cured tobacco (*Nicotiana tabacum* L.). Plant Breed.

[40] Goutam U, Kukreja S, Yadav R, Salaria N, Thakur K, Goyal AK. Recent trends and perspectives of molecular markers against fungal diseases in wheat. Front Microbiol. 2015;6:861. 10.3389/fmicb.2015.00861 PMC4548237 26379639

[41] Miah G, Rafii M, Ismail M, Puteh A, Rahim H, Islam K, Latif M (2013). A review of microsatellite markers and their applications in rice breeding programs to improve blast disease resistance. Int J Mol Sci.

[42] Mir R, Zaman-Allah M, Sreenivasulu N, Trethowan R, Varshney R (2012). Integrated genomics, physiology and breeding approaches for improving drought tolerance in crops. Theor Appl Genet.

[43] Serba D, Wu L, Daverdin G, Bahri B, Wang X, Kilian A, Bouton J, Brummer EC, Saha M, Devos K (2013). Linkage maps of lowland and upland tetraploid switchgrass ecotypes. Bioenerg Res.

[44] Yu J, Dossa K, Wang L, Zhang Y, Wei X, Liao B, Zhang X. PMDBase: a database for studying microsatellite DNA and marker development in plants. Nucleic Acids Res. 2016;45(D1):D1046–D1053. 10.1093/nar/gkw906. 10.1093/nar/gkw906 PMC5210622 27733507

[45] Beier S, Thiel T, Munch T, Scholz U, Mascher M. MISA-web: a web server for microsatellite prediction. Bioinformatics. 2017;33(16):2583–85. 10.1093/bioinformatics/btx198 PMC5870701 28398459

[46] Schuler GD (1997). Sequence mapping by electronic PCR. Genome Res.

[47] Camacho C, Coulouris G, Avagyan V, Ma N, Papadopoulos J, Bealer K, Madden TL (2009). BLAST+: architecture and applications. BMC Bioinf.

