# Supplementary material for: Comparative genome-wide characterization leading to simple sequence repeat marker development for Nicotiana
Source: BMC Genomics. 2018 Jun 27;19:500. doi: 10.1186/s12864-018-4878-4 (PMC6020451; doi:10.1186/s12864-018-4878-4)
Supplement: Supplementary file 2 — Figure S1 SSR length distribution. (PDF 364 kb) [file 12864_2018_4878_MOESM2_ESM.pdf]

Supplementary material: figure

Comparative genome-wide characterization leading to simple sequence repeat marker development for *Nicotiana*

Xuewen Wang[1,2], Shuai Yang[3], Yongdui Chen[4], Shumeng Zhang[2], Qingshi Zhao[1], Meng Li[1], Yulong Gao[5], Long Yang[3]*, Jeffrey L. Bennetzen[1,2]*

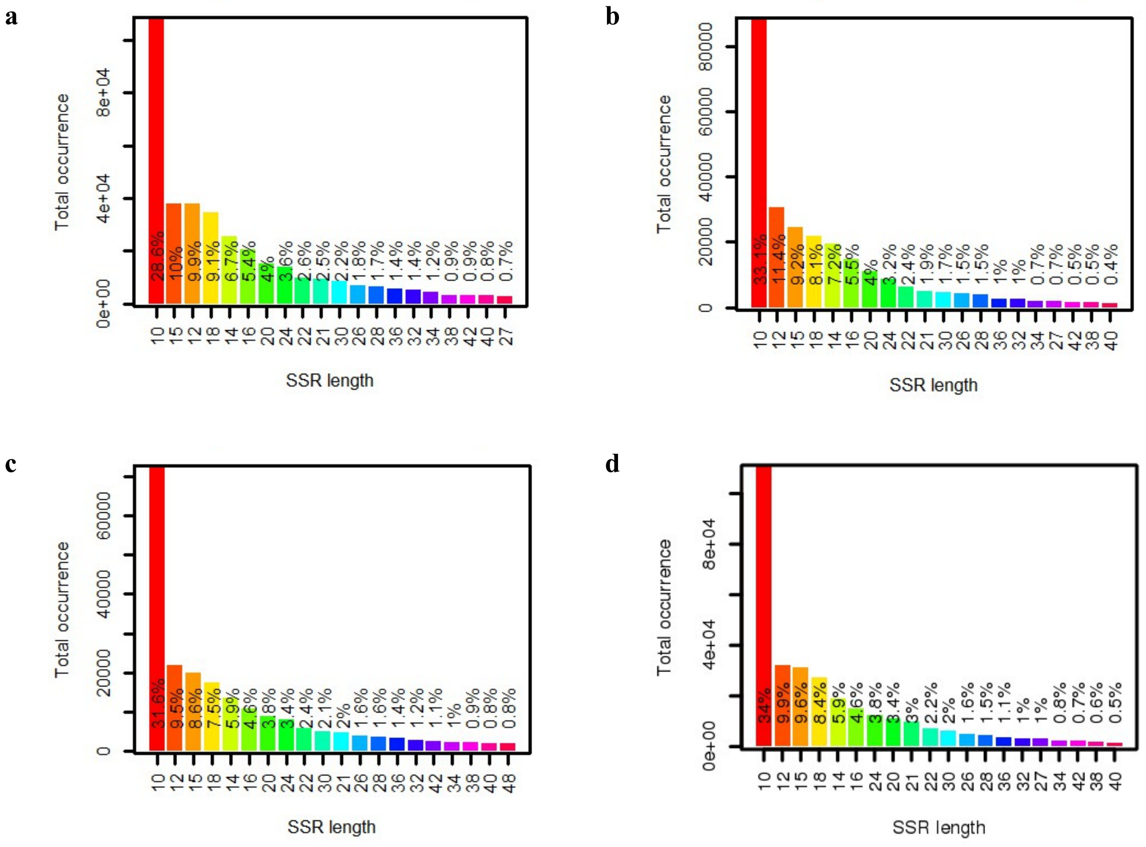

e

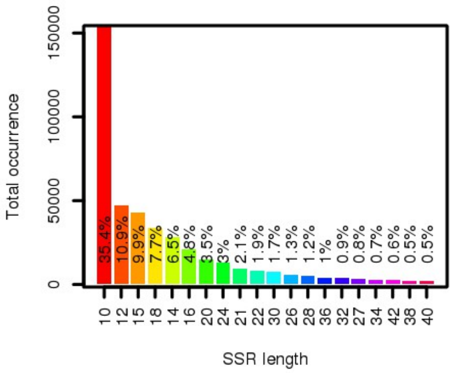

f

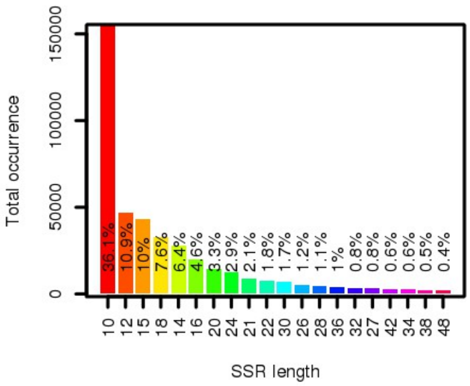

g

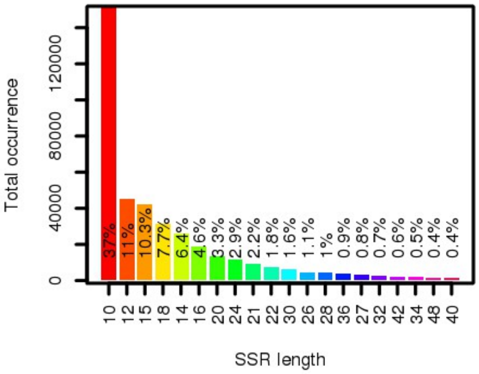

Figure S1. Length distributions of SSRs in *Nicotiana* species

a, b, c, d, e, f and g represent the distribution of the top 20 SSR lengths in *N. ben*, *N. syl*, *N. tom*, and *N. oto*, and *N. tab* TN90, K326 and BX. Total occurrence is ordered from highest (left) to lowest (right).
